# A new species of *Haplothrips* from southern Iran (Thysanoptera, Phlaeothripidae)

**DOI:** 10.3897/zookeys.275.4433

**Published:** 2013-03-06

**Authors:** Kambiz Minaei, Maryam Aleosfoor

**Affiliations:** 1 Department of Plant Protection, College of Agriculture, Shiraz University, Shiraz, Iran

**Keywords:** *Haplothrips*, new species, phytophagous, *Suaeda* sp.

## Abstract

*Haplothrips herajius* **sp. n.** is described from leaves and flowers of a species of *Suaeda* in the south of Fars Province, Iran. This is the second Iranian species of *Haplothrips* with the unusual character state of extra setae on the metanotum. Information on variation in color and structure of the new species is provided. The similarities and host plant associations of this new species and *Haplothrips kermanensis* are discussed,as both are phytophagous on species of Chenopodiaceae.

## Introduction

Traditionally and still widely accepted, the known species of thrips are placed in a single order, the Thysanoptera, within which two suborders are recognized, the Terebrantia and Tubulifera (Mound et al. 1980, Mound and Morris 2007, Buckman et al. 2013). The suborder Tubulifera comprises a single family, the Phlaeothripidae with about 3500 described species (Mound 2013) classified into two subfamilies. In the more speciose of the subfamilies, the Phlaeothripinae, *Haplothrips* Amyot & Serville, with 226 species worldwide, is the second largest genus, exceeded in number of described species only by *Liothrips* Uzelwith 250 species (Mound 2013). Most species in this genus are Eurasian with just three described from South America (Mound and Zapater 2003, see also Goldarazena et al. 2012) and some world-wide in distribution (Pitkin 1976).

Among 20 genera of Phlaeothripidae recorded from Iran so far (Minaei 2013), the genus *Haplothrips* is considered to be the richest in this country (Minaei and Mound 2008). Apart from some species that are predators on other arthropods (Putman 1965, Bailey and Caon 1986, Palmer and Mound 1990, zur Strassen 1995, Kakimoto et al. 2006, Okajima 2006) most species in this genus live on two plant families, Asteraceae and Poaceae, with a few species found on plants in other families. One of these is *Haplothrips kermanensis* zur Strassen which was described from Iran based on specimens collected on *Haloxylon* sp. (Chenopodiaceae) (zur Strassen 1975), and this appears to be a specific host for this thrips (Minaei and Mound 2008). The objective of this paper is to describe a new species of *Haplothrips* collected on another chenopod species, *Suaeda* sp. These two thrips species are very similar in structure as discussed below.

## Materials and methods

The new species discussed below was collected by beating leaves and flowers of *Suaeda* sp. (Chenopodiaceae) onto a plastic tray. The specimens were removed with a fine brush into a collecting vial containing 90% ethyl alcohol. They were then mounted onto slides in Canada balsam using a form of the protocol given by Mound and Kibby (1998). The line drawings were sketched using a drawing attachment. Terminology follows Mound and Minaei (2007) and Minaei and Mound (2008). The holotype and other specimens studied here are deposited in the collection of the Department of Plant Protection, College of Agriculture, Shiraz University, Shiraz, Iran. A few paratypes are deposited in the Australian National Insect Collection, Canberra and the Natural History Museum, London. The following abbreviations are used for pronotal setae: am—anteromarginals; aa—anteroangulars; ml—midlaterals; epim—epimerals; pa—posteroangulars.

## Taxonomy

### Haplothrips herajius

sp. n.

urn:lsid:zoobank.org:act:FFCC20A9-BDC2-4700-B252-C87F008BA82B

http://species-id.net/wiki/Haplothrips_herajius

#### Type material.

Holotype female, Iran, Fars Province, Mohr, Heraj village; *Suaeda* sp. (leaves), 31.iii.2012. (Mohsen Abdolahi); Paratypes: 58 females, 11 males taken with holotype; 14 females, 3 males, same place, *Suaeda* sp. (flowers), 21. ix. 2012.

#### Description.

Female macroptera. Body brown (paler in summer forms), all tarsi, fore tibiae in distal half, distal apex of mid and hind tibiae are yellow; antennal segments I–II brown but the color of remaining segments variable depending on collecting date (III–VI yellow, VII–VIII yellow-brownish in summer forms; III yellow, IV–VIII yellow-brownish, gradually darker brown in spring forms); fore wing pale except for basal area; major body setae as well as sub-basal wing setae pale but tergite setae and anal setae slightly shaded at base.

**Figures 1–9.**
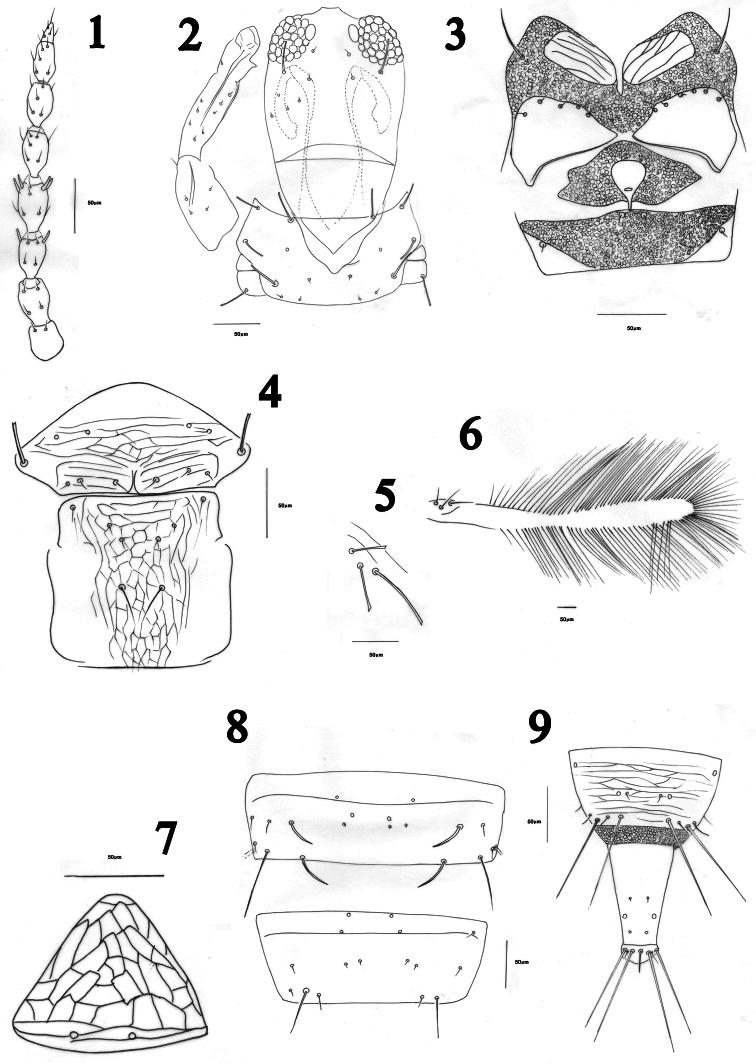
*Haplothrips herajius* sp. n. Female. **1** Antenna **2** Head and pronotum **3** Prosternum and mesopresternum **4** Mesonotum and metanotum **5** Sub basal wing setae **6** Forewing **7** Pelta **8** Tergites VII-VIII **9** Tergite IX and tube.

Antennae 8-segmented, segment III with two, IV with four sensoria, VII slightly constricted at base, VIII short and broad at base (Fig. 1). Head a little longer than wide with maxillary stylets 0.2–0.3 of head width apart, retracted anterior to post ocular setae; post ocular setae blunt or capitate, extending to posterior margin of eye (Fig. 2). Cheeks weakly rounded. Maxillary bridge well developed. Mouth cone rounded.

Pronotum transverse, without sculpture lines except close to posterior margin; notopleural sutures complete; five pairs of developed setae present: am, aa, ml, epim and pa, all blunt or capitate (Fig. 2); prosternum with paired basantra and ferna as well as a spinasternum, ferna broad (Fig. 3). Mesonotum transversely weakly reticulate, with no microtrichia, lateral setae well developed, weakly capitate (Fig. 4). Mesopresternum eroded medially (Fig. 3). Metanotum reticulate, with no microtrichia, median setae slender and acute, arise on posterior half of sclerite, with 2–4 small setae on anterior half (Fig. 4). Fore tarsal tooth conspicuous (Fig. 2). Fore wing constricted medially (Fig. 6), sub-basal setae S1, S2 and S3 blunt or capitate, their bases arranged in a triangle (Fig. 5), 2–7 duplicated cilia present (Fig. 6).

Pelta triangular, weakly reticulate (Fig. 7). Tergite II–VII with wing-retaining setae, anterior pair weaker than posterior one, these being weakest on tergite II; tergites II–VII with a few lines of sculpture and 3–5 discal setae lateral to two pairs of developed wing-retaining setae; marginal setae S1 and S2 on tergites VII–IX long and finely pointed, S2 on other tergites finely pointed but S1 usually blunt, rarely finely pointed and sometimes variable on different tergites, tending to be more pointed on posterior than anterior tergites. Tergite VII with two campaniform sensilla not close to each other, separated by at least 0.1 width of tergite, with four micro-setae laterally; tergite VIII campaniform sensilla further apart, more than two times as those on tergite VIII, three to four micro-setae between sensilla or sometimes in front of them (Fig. 8). Tube short, about twice as long as basal width (Fig. 9); anal setae usually longer than tube.

Measurements.(holotype female, in microns). Body distended length 1845. Head, length 190; median width 180; postocular setae 35. Pronotum, length 35; width 68; major setae am 34, aa 33, ml 26, epim 51, pa 43. Fore wing length 700; sub-basal wing setae 41, 50, 68. Tergite IX setae S1 95, S2 85. Tube length 108; basal width 58. Antennal segments III–VIII length 38, 47, 43, 41, 33, 21.

**Figures 10–11.**
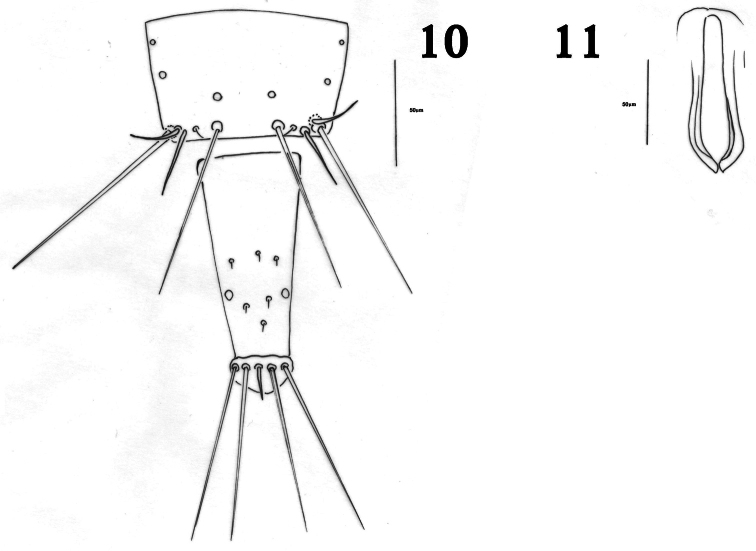
*Haplothrips herajius* sp. n. Male **10** Tergite IX and tube **11** Pseudovirga.

Male macroptera. Color and structure similar to female. Sternites with no pore plates; tergite IX setae S2 short and stout (Fig. 10). Pseudovirga spoon shaped at apex (Fig. 11).

#### Diagnosis.

**Figures 12–14.**
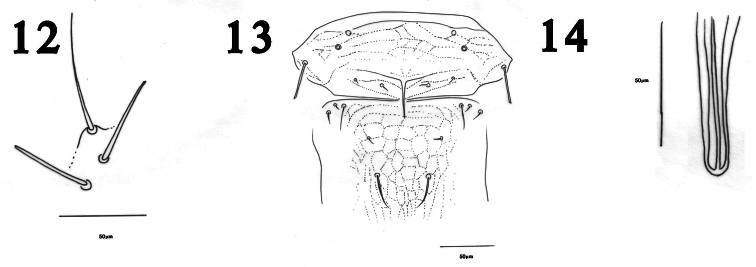
*Haplothrips kermanensis*.Female **12** Sub basal wing setae **13** Mesonotum and metanotum. Male **14** Pseudovirga.

The reticulation on the mesonotum and metanotum of *Haplothrips herajius* (Fig. 4) is unique among Iranian *Haplothrips* as well as for most other *Haplothrips* species. In other species of *Haplothrips* recorded from Iran, this reticulation is weakly developed or absent. The new species is very close to *Haplothrips kermanensis*. Both species have extra setae on metanotum (Figs 4, 13) that are not seen in other Iranian species of *Haplothrips*. Moreover, in both species the basal wing setae are arranged in a triangle (Figs 5, 12) (this arrangement in *Haplothrips kermanensis* was not reported by Minaei and Mound (2008), and the apex of the mid and hind tibiae are pale, also a conspicuous fore tarsal tooth is present in both species. However, the number of small setae anterior to the median pair of metanotal setae in the new species is variable, 2–4 (rarely 0, 5 or 6), whereas available specimens of *Haplothrips kermanensis* all consistently have one pair. In addition, am setae on the pronotum in *Haplothrips herajius* are blunt or capitate in contrast to *Haplothrips kermanensis* in which they are pointed. Furthermore, fore wing sub-basal setae S3 in the new species is blunt compared with weakly pointed in *Haplothrips kermanensis*. Males of the two species are clearly different in genitalia: spoon shaped in *Haplothrips herajius* but rod shaped in *Haplothrips kermanensis* (Figs 11, 14).

#### Variability.

Color of body and antennal segments varies among specimens, being paler in summer specimens compared with specimens collected in early spring. The fore tarsal tooth is conspicuous, but variable from small to large among male specimens. Maxillary stylets are retracted to postocular setae but rarely are low in the head and not reaching the postocular setae. Moreover, in a few specimens, the pronotal am setae are not developed.

#### Etymology.

Heraj is a village of Mohr city in the south of Fars Province, south of Iran which is located 300 km south of Shiraz, the capital of Fars Province.

## Discussion

The presence on two separate chenopod species of two *Haplothrips* species that share unusual character states, as discussed above, is interesting. The large number of collected specimens of *Haplothrips herajius* on both leaves (early spring) and flowers (late summer) of *Suaeda* sp. suggest that the new species is phytophagous, and apparently this plant species is a specific host for the new species in that area. Recently Minaei et al. (2012) described another thrips species, *Ankothrips zayandicus* (Melanthripidae), from the same plant in Isfahan Province, central Iran.

Considering that most species of Chenopodiaceae bloom in summer months, the specimens studied here were collected at two seasons: in early spring on leaves and in late summer on flowers. The color of body and antennae differs between specimens collected at these two seasons as mentioned above. This difference is remarkable because the effect of temperature on body color during development was not noticed in any species of *Haplothrips* so far. However, in onion thrips, *Thrips tabaci*, a well-known pest of thripid family, experiments showed low temperatures during pupal development induce dark adult body color (Murai and Toda 2002). Similarly, in Australia an endemic and very common thrips, plague thrips, *Thrips imaginis*, is commonly dark after winter but pale yellow during summer (Mound and Masumoto 2005).

Bhatti et al. (2009) in their book on the Thysanoptera of Iran listed 29 species of *Haplothrips*, among them two species (*Haplothrips bagnalli*, *Haplothrips* nr. *bagrolis*) reported by Manzari (2004) from Iranian islands.However, only one *Haplothrips* (*Haplothrips bagnalli*) was reported by him (the other two species were thripids), although towards the end of the report he introduces, obscurely, a comparison between *Haplothrips* nr *bagrolis* and *Haplothrips ganglbaueri*. The somewhat cursory report was published in an informal newsletter and, given its nature; the presence of both species in Iran requires further verification. Similarly, the report of three other species, *caespitis*, *minutus* and *rabinovitchi* by Bagheri and Alavi (2007) needs to be confirmed (Minaei and Mound 2008, Bhatti et al. 2009).Moreover, Minaei and Mound (2010) demonstrated that *Haplothrips cerealis* does not occur in Iran, the reported occurrence of this species being a misidentification of *Haplothrips tritici*. So with the addition of the species described here, the confirmed Iranian species in *Haplothrips* comprise 24 species i.e. about 12% of the total Thysanoptera fauna in this country. Similarly this genus comprises about 8% of Thysanoptera fauna in Britain (Collins 2010). In contrast, the species composition of the Australian insect fauna (southern Hemisphere) is very different and the species of *Haplothrips* comprise about 3% of the known thrips fauna of that continent (Mound and Minaei 2007).

## Supplementary Material

XML Treatment for Haplothrips herajius
